# Supplementary material for: Resolving the full spectrum of human genome variation using Linked-Reads
Source: Genome Res. 2019 Apr;29(4):635–45. doi: 10.1101/gr.234443.118 (PMC6442396; doi:10.1101/gr.234443.118)
Supplement: Supplemental Material [file supp_gr.234443.118_Supplemental_longranger-2.2.zip › longranger-2.2.tar.bz2!/lr2.2-public/lib/stan/cmdstan/stan/doc/api/html/operator__unary__plus_8hpp.html]

Stan: src/stan/agrad/rev/operators/operator\_unary\_plus.hpp File Reference


|  |  |
| --- | --- |
|  | Stan  2.5.0  probability, sampling & optimization |


- Main Page
- Related Pages
- Namespaces
- Classes
- Files

- File List
- File Members

All Classes Namespaces Files Functions Variables Typedefs Enumerations Enumerator Friends Macros Pages

- src
- stan
- agrad
- rev
- operators

Namespaces |
Functions

operator\_unary\_plus.hpp File Reference

`#include <stan/agrad/rev/var.hpp>`  
`#include <boost/math/special_functions/fpclassify.hpp>`  
`#include <stan/agrad/rev/internal/precomp_v_vari.hpp>`  
`#include <stan/math/constants.hpp>`

Go to the source code of this file.

|  |  |
| --- | --- |
| Namespaces | |
|  | stan |
|  | Probability, optimization and sampling library. |
|  | |
|  | stan::agrad |
|  | Function gradients via reverse-mode automatic differentiation. |
|  | |

|  |  |
| --- | --- |
| Functions | |
| var | stan::agrad::operator+ (const var &a) |
|  | Unary plus operator for variables (C++). More... |
|  | |


---

[ Stan Home Page ]

*© 2011–2014,
Stan Development Team.*
